# Understanding the Impact of Algorithmic Discrimination on Unethical Consumer Behavior

**DOI:** 10.3390/bs15040494

**Published:** 2025-04-08

**Authors:** Binbin Sun, Shan Pei, Qingjin Wang, Xuelei Meng

**Affiliations:** School of Business, Qingdao University, Qingdao 266071, China; iklovey@163.com (B.S.); peishan@qdu.edu.cn (S.P.); mengxl1230@163.com (X.M.)

**Keywords:** unethical consumer behavior, anticipatory guilt, negative reciprocity, social exchange theory, moral decisions, human–AI interaction, artificial intelligence agent

## Abstract

The prevalence of artificial intelligence (AI) increases social concern surrounding unethical consumer behavior in human–AI interaction. Existing research has mainly focused on anthropomorphic characteristics of AI and unethical consumer behavior (UCB). However, the role of algorithms in unethical consumer behavior, which is central to AI, is not yet fully understood. Drawing on social exchange theory, this study investigates the impact of algorithmic discrimination on UCB and explores the interrelationships and underlying mechanisms. Through three experiments, this study found that experiencing algorithmic discrimination significantly increases UCB, with anticipatory guilt mediating this relationship. Moreover, consumers’ negative reciprocity beliefs moderated the effects of algorithmic discrimination on anticipatory guilt and UCB. In addition, this study distinguish between active and passive UCB based on their underlying ethical motivations. This enhances the study’s universality by assessing both types of behaviors and highlighting their differences. These insights extend current research on UCB within the purview of AI agents and provide valuable insights into effectively mitigating losses caused by UCB behaviors, offering improved directions for facilitating AI agents to provide fair, reliable, and efficient interactions for both businesses and consumers.

## 1. Introduction

With the development of artificial intelligence (AI) technology, AI has been integrated into all aspects of life, which can not only bring profits to enterprises but also bring convenience to people’s daily life. However, it remains an open and immature field (Guan et al., 2022). AI’s personalized services boost company profits, but unethical customer behavior (UCB) in human–computer interactions incurs costs (Babakus et al., 2004). Studies show that consumers interacting with AI agents may have less anticipatory guilt resulting in more UCB, such as theft from unmanned stores or abuse of AI assistants (Giroux et al., 2022; Kim et al., 2023).

Algorithms are the logic behind AI, and much discrimination in real life has evolved into algorithm discrimination with the development of AI. Algorithm discrimination has become common in our life, from job searches to shopping (Z. Chen, 2023). Many users are becoming aware of algorithmic opacity and experiencing discrimination (Someh et al., 2019). Responses to consumer behavior resulting from algorithmic discrimination include rejecting algorithmic recommendations (Z. Chen, 2023), having an aversion to algorithm participation (Dietvorst et al., 2018), and rejecting algorithmic systems (Lee et al., 2019). Some scholars also point out that algorithmic discrimination may provoke fewer negative emotions than human discrimination (Bigman et al., 2023). However, the impact of algorithmic discrimination on UCB remains to be explored.

In addition, when combing the literature, some differences in questionnaires when measuring UCB were found. Specifically, some scholars described the active UCB scenario in the questionnaire (Kim et al., 2023), while others used the passive UCB scenario (Giroux et al., 2022). This study argues that there are significant differences between these two types of unethical consumer behavior: First, people have different moral judgments about active and passive unethical behavior. Consumers may believe that if they are not initiating the unethical activity, it is less wrong (Vitell, 2003). Compared to active unethical behavior, passive unethical behavior is perceived as less immoral, and the psychological burden brought to people is lower. These judgments about the degree of unethical behavior influence consumers’ engagement in such behavior (Akaah, 1989; Hunt & Vitell, 1986; Robin et al., 1996). Second, companies respond differently to these two types of UCB. Passive UCB is consumers passively profiting from errors in AI agents. Firms can reduce this by enhancing technology to minimize errors. In contrast, active UCB is consumers intentionally engaging in unethical acts for personal gain. Addressing active UCB requires enterprises to improve the ability of AI agents to identify errors. Therefore, it is necessary to analyze the two unethical behaviors differently in the research design.

Nowadays, more and more enterprises use AI agents to provide services to consumers. For example, Alibaba Group’s FlyZoo Hotel uses AI service robot to provide check-in/checkout and other services. In addition, many enterprises gradually provide self-checkout and algorithm-based data analysis services, which can not only bring considerable benefits to enterprises and save long-term costs but also improve the consumer service experience (Davenport et al., 2020). This study is mainly based on the service sector, exploring the impact of algorithmic discrimination on the UCB and aiming to provide constructive suggestions for enterprises to better use artificial intelligence agency.

## 2. Literature Review

### 2.1. UCB

The extensive marketing literature discusses UCB, encompassing a spectrum of actions from minor infractions, such as misrepresenting one’s lack of qualification for a discount, to severe and illicit activities like shoplifting or perpetrating deceitful returns (Y. Chen et al., 2023; V. W. Mitchell et al., 2009). Fullerton and Punj characterize UCB as actions that contravene ethical standards or moral guidelines in the consumer sphere, inflicting damage on the brand or fellow customers (Fullerton & Punj, 1993). Such behaviors may encompass deceit, fraud, vindictive returns, theft, and similar actions (Fullerton & Punj, 2004). Vitell and Muncy distinguish UCB into four categories: engaging in illegal actions for personal gain (such as altering price tags), passively profiting from unlawful actions (like failing to report over-given change), actively taking advantage of legally dubious activities (such as utilizing expired vouchers), and non-harmful, unintentional acts (like extended clothing trials without purchase) (Vitell & Muncy, 1992). It is worth mentioning that Mayr et al. systematically investigated and summarized the causes of UCB, its specific performance, and its impact on frontline employees and customers in the context of retail stores (Mayr et al., 2022). These studies can help us to better understand the concept and scope of UCB.

Numerous scholars have proposed various models to explain UCB, with some focusing on individual characteristics. For instance, a lack of intrinsic motivation linked to materialism may lead to unethical behavior (Arli & Tjiptono, 2014). Moreover, people who possess high self-monitoring and moral standards, or those with low Machiavellian tendencies, are less inclined to participate in unethical actions (Wirtz & Kum, 2004; Xiong et al., 2023). Zhao explore the relationship between an individual’s place of birth and UCB (Zhao & Xu, 2013). In addition, various environmental elements can shape UCB, such as the degree of social approval (Mills & Groening, 2021), disparities in resource allocation and social interaction (Wirtz & McColl-Kennedy, 2010), and experiences of service exclusion (Gong et al., 2022).

In order to improve the quality of frontline services, more and more service providers are using AI service robots to replace human employees (McLeay et al., 2021). However, some scholars have pointed out that some characteristics of AI agents enable people to pursue unethical interests while maintaining a good moral image (Köbis et al., 2021). Giroux et al. show that consumers tend to behave more unethically when they encounter AI agents (Giroux et al., 2022; Kim et al., 2023). This means that using AI service agents does not necessarily reduce operating costs for the enterprise but may increase UCB and thus lead to additional losses. This issue caused a widespread research frenzy as soon as it was pointed out. LaMothe pointed out that individuals are more likely to lie to cold machines than to humans (LaMothe & Bobek, 2020). Mubin et al. also showed that people show more cheating when facing robots (Mubin et al., 2020; Petisca et al., 2020).

Subsequently, many scholars explored the factors that affect UCB in the context of using AI agents. For example, Lee and other scholars started from the type of AI agents and found that consumers show more unethical behavior when facing servant AI agents than partner AI agents (Lei et al., 2024). Li pointed to the impact of AI identity disclosure on UCB. They found that disclosure of AI agents led more UCB compared to no disclosure (Li et al., 2024). In addition, some scholars have studied the characteristics of consumers and pointed out that when interacting with service robots that communicate in a polite way, consumers with independent self-construct (vs. interdependent self-construct) show a higher intention to engage in unethical behavior (Dong et al., 2025). Unlike the previous consensus that using AI agents would increase UCB, Liu et al. explored the action boundaries of different agents on UCB. They point out that in the case of service inclusion, consumers will engage in more UCB for AI agents, but in the case of service exclusion, the situation changes, and consumers are more likely to engage in UCB for human agents (Y. Liu et al., 2023).

However, in the process of combing the literature, this study found that the questionnaire used by the scholars were different when measuring UCB. Kim and Zhao used the active UCB scenario as questionnaire material, such as hiding mistakes for higher insurance payouts or using expired coupons (Kim et al., 2023; Zhao et al., 2020). Giroux and other scholars focused on passive UCB, where consumers do not report billing errors in their favor (Giroux et al., 2022). The primary difference between active and passive UCB lies in who is responsible for the unethical behavior—buyer or seller. Active UCB involves consumers actively seeking benefits at the seller’s expense, like altering price tags or using expired coupons. Passive UCB occurs when consumers passively benefit from the seller’s mistakes, such as receiving too many deals without speaking up. Traditionally, passive benefitting is seen as more ethical than actively seeking undeserved benefits (Vitell, 2003). This paper considers that it is necessary to test these two types of UCB, which can improve the comprehensiveness and persuasion of the study.

### 2.2. Algorithmic Discrimination

Algorithmic discrimination refers to the unfair treatment of specific groups or individuals during the design, training, or use of algorithms, stemming from issues with the data or the algorithms themselves (Favaretto et al., 2019). Such inequity can manifest as bias, discriminatory decision-making, or differential treatment of specific groups, distinguished based on various factors including gender, ethnicity, age, geographic location, and more. For example, there is a lot of gender discrimination in recruitment algorithms, as seen at Amazon, where men dominate management roles, reflecting the system’s gender bias. In the consumer market, algorithmic discrimination is obvious in terms of price. For example, the dynamic pricing strategy of airlines may lead to different fares for the same flight for different users (Moreno-Izquierdo et al., 2015).

With the development of artificial intelligence, many scholars have made comparative studies on the discrimination between humans and algorithms. Many psychological studies have found that even if AI makes the same level of immoral decisions as humans, people will show more tolerance of AI, a phenomenon that researchers call the artificial intelligence moral deficit effects (Bigman et al., 2023; Wilson et al., 2022). Some scholars have discussed the differences in the level of anger that people have in the face of sexist decisions made by HR experts and AI recruitment processes. The results showed that algorithmic discrimination caused less feelings of anger than discrimination from humans (Bigman et al., 2023). In terms of behavior performance, compared with human discrimination, people will also have less opposing action and punishment tendency for algorithmic discrimination. Research by XU and other scholars shows that people show less moral desire to discriminate against algorithms than humans (Xu et al., 2022). The above study is designed to compare people responding to discrimination from different agents (human vs. algorithms). Different from previous research, scholar Ghasemaghaei discusses unethical behavior in the face of algorithmic discrimination. The findings suggest that decision makers may make discriminatory decisions with less guilt under the influence of algorithmic discrimination recommendations. This study suggests that algorithmic discrimination may be a catalyst to aggravate people’s unethical behavior (Ghasemaghaei & Kordzadeh, 2024). In conclusion, previous scholars have explored the relationship between algorithmic discrimination and emotions such as anger and guilt and explored the influence of algorithmic discrimination on people’s behavior. Some scholars have pointed out that the use of AI agents may lead to the rise of UCB, so it remains to be studied whether consumers will feel less guilt to make more UCB for AI agents or show a tolerant attitude towards algorithm discrimination.

## 3. Hypothesis Development

### 3.1. Algorithmic Discrimination, Anticipatory Guilt, and UCB

Frequently, the social exchange theory is invoked to account for people’s unethical behavior (Gong et al., 2022; Y. Wang et al., 2024). Among the core concepts of this theory is the principle of reciprocity, which includes both positive and negative aspects (Zhu et al., 2023). Positive reciprocity involves returning help, while negative reciprocity involves retaliating for harm (Perugini et al., 2003). The principle of negative reciprocity is that if the provider harms the recipient, the latter will retaliate against the former (Cropanzano & Mitchell, 2005; Eisenberger et al., 2004). Retaliation is considered an appropriate response to bad behavior, functioning to maintain equilibrium and ensure fair interpersonal exchanges within the social system (Y. Chen et al., 2009). This paper focuses on the influence of algorithmic discrimination on UCB, which is an unfair negative behavior from algorithms. Therefore, this paper focuses on the negative reciprocity principle in social exchange theory. This study argue that, based on the principle of negative reciprocity, consumers who experience algorithmic discrimination may retaliate and thus make more UCB for AI agents. Therefore, this study propose the following hypothesis:

**H1.**  *After experiencing algorithmic discrimination, consumers are more likely to engage in UCB towards AI agents.*

Guilt occurs when one thinks their actions cause harm to others (Meque et al., 2023; Scaffidi Abbate et al., 2022). Research indicates that an individual’s moral decision-making is closely tied to emotions (Schein & Gray, 2015). Guilt influences future behavior and is felt when individuals realize that their actions violate ethical standards (Baumeister et al., 2007; Choi, 2023, 2024; Saintives, 2020). Some scholars have pointed out that guilt is an important factor to restrain unethical behavior (Tibbetts, 2003).

Guilt types include anticipatory, reactive, and presence guilt. Studies indicate that the feeling of anticipatory guilt can prevent people from partaking in actions that could lead to guilt (Baumeister et al., 1994). When faced with a moral dilemma, individuals may experience anticipatory guilt, a moral emotion triggered by the expectation of acting immorally (Choi, 2022). Mills have shown a negative correlation between anticipatory guilt and behaviors like pirating movies (Mills & Groening, 2021). Mazar et al. found that a rise in anticipated guilt reduced the likelihood of lying for personal gain (Mazar et al., 2008).

In our theoretical framework, algorithmic discrimination is viewed as a biased negative behavior, consistent with the principle of negative reciprocity. Therefore, consumers harmed by the AI agent may retaliate against the AI agent. Driven by the revenge mentality, this study speculate that after consumers encounter algorithmic discrimination, when they fall into a moral dilemma when facing AI agents, their anticipatory guilt decreases and they are more likely to commit unethical behavior towards AI agents. Based on this view, this study propose the following hypothesis:

**H2.**  *After experiencing algorithmic discrimination, consumers will feel less anticipatory guilt towards AI agents when confronted with them.*

**H3.**  *Anticipatory guilt mediates the effect of algorithmic discrimination on UCB.*

### 3.2. The Moderating Role of Negative Reciprocity Beliefs

The negative reciprocity principle suggests that people will retaliate for perceived harm (Y. Chen et al., 2009; Eisenberger et al., 2004). Each individual has varying levels of negative reciprocity beliefs: those with strong beliefs are more likely to retaliate when feeling victimized, while those with weaker beliefs may choose reconciliation, avoidance, or forgiveness (Gong et al., 2022; Eisenberger et al., 2004; J. Liu et al., 2010). Eisenberger and other scholars also have determined that individuals with strong negative reciprocity beliefs exhibit a heightened propensity for retribution (Eisenberger et al., 2004).

Based on the social exchange theory, this study explores how algorithmic discrimination affects UCB. This study guess that, after experiencing discrimination, those consumers with stronger negative reciprocal beliefs were more likely to make more UCB with lower expected guilt, and those with weaker negative reciprocal beliefs will have a more moderate response. Thus, this study proposes the following hypotheses concerning the linkage between negative reciprocity beliefs, algorithmic discrimination, anticipatory guilt, and UCB:

**H4a.**  *Negative reciprocity beliefs moderated the effect of algorithmic discrimination experiences on UCB.*

**H4b.**  *Negative reciprocity beliefs moderated the effect of algorithmic discrimination experiences on anticipatory guilt.*

**Figure 1:**
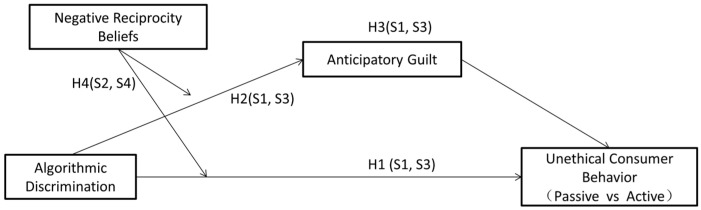
Research model diagram.

In summary, the model diagram for this study is shown in Figure 1.

## 4. The Overview of the Research

### 4.1. Pre-Experimentation

This experiment pre-tested stimulus material for algorithmic discrimination with 80 subjects (61.3% female, Age concentration 26–35 years) recruited via Credamo. All questionnaires in this study were distributed and collected on Credamo, a platform for large-scale online research, where the data collected have been adopted in numerous papers. Specific details of the relevant demographic variables can all be viewed in Appendix A. Details of the questionnaires for all experiments can be found in Appendix B. They were randomly divided into three groups: algorithmic discrimination, active UCB, and passive UCB. Subjects in the algorithmic discrimination group were asked to read a passage, imagining they had been discriminated against by the restaurant’s AI agent, and then answer questions. Subjects rated perceptions of perceived discrimination (PD) and perceived anger (PA) on a sliding scale from 0 (completely disagree) to 100 (completely agree) (see Appendix B for specific questions). The title item was adapted from the study by Russell (Russell & Giner-Sorolla, 2011). The results indicated that the manipulation of algorithmic discrimination was successful (M_PD_ = 84.77, M_PA_ = 86.79), both at high levels, suggesting that the material was suitable for use in a formal experiment.

Then, subjects were assigned to active or passive UCB groups to evaluate the realism, clarity, and immersion of our UCB scenario descriptions. After reading the scenarios described in the questionnaire, subjects answered three questions on a five-point scale about the scenario’s similarity to reality (‘How similar do you think the above-described scenario is to reality?’), clarity (‘What do you think is the clarity of the scenario described above?’), and sense of presence (‘Do you think the scenario described above is something you could put yourself in?’). Analysis showed high scores for reality similarity (M = 4.41), clarity (M = 4.43), and immersion (M = 4.43), suggesting the experiment effectively manipulated these aspects and subjects found the material realistic and engaging.

### 4.2. Experiment 1: Algorithmic Discrimination, Anticipatory Guilt, and Passive UCB

In the previous section, this study pointed out that when measuring UCB, some scholars used active UCB scenarios in their questionnaires, while others used passive UCB scenarios. Previous studies have shown that it is more unethical to actively seek illegitimate benefits for oneself than to passively benefit from them (Vitell & Muncy, 1992; Vitell, 2003), and that thought may inhibit their likelihood of engaging in active UCB. This paper focuses on the effect of algorithmic discrimination on UCB, so it is necessary to fully validate these two different types of UCB to ensure the reliability of the article’s conclusions. The questionnaires for Experiments 1 and 2 described passive UCB situations, while Experiments 3 and 4 used active UCB situations. In Experiment 1, this study referred to the Giroux study and described a restaurant service scenario (Giroux et al., 2022). The restaurant is a consumption scene that consumers are very familiar with, and such a scene description is more conducive to the participants to bring them in better. Experiment 1 shows that algorithmic discrimination increases passive UCB and reduces anticipatory guilt when interacting with AI, as predicted.

#### 4.2.1. Method

This experiment recruited subjects via Credamo and provided them with some compensation. The platform has a large number of users and can widely release questionnaires, and the questionnaire quality is high. The subjects were randomized into two experimental groups: one with algorithmic discrimination and one without. In this round and the following experiments, we ensured the randomness of the experimental questionnaire issuance in the following ways: First, this study used the randomization tool provided by this platform, which ensured that subjects in the questionnaire were assigned into any experimental group with equal probability. Second, the platform adopts the real-name registration method, and this study prohibited repeated answers to the same users, to ensure that the same subject can only participate in one experiment. Finally, for the collected questionnaires, each round of experiments needed to analyze the demographic variables of each group in order to test whether there are significant differences between the subjects in the two groups. If there is no significant difference in their demographics, it can also indicate that the subjects were randomly assigned into different experimental groups.

Subjects in the algorithmic discrimination group will first read the same passage of algorithmic discrimination material as in the pre-experiment and answer questions related to perceptions of discrimination and anger. Afterward, they read UCB material similar to the non-discrimination group, based on scenes from Bateman and Valentine (Bateman & Valentine, 2010). Subjects were instructed to envision themselves at a local restaurant where, received a bill in their favor (a bill that was originally JPY 260 was incorrectly calculated as JPY 220).

To address the sensitivity of the ethical scenario, a virtual protagonist named Zhang San was used in the experimental scenario. After reading the material, subjects answered a UCB-related question referenced from Giroux’s study (Giroux et al., 2022), which asked, ‘If you were Zhang San, would you do this?’ Subjects indicated their UCB intentions on a 7-point scale (1 = strongly disagree, 7 = strongly agree). Subsequently, subjects were asked to assess their anticipatory guilt for engaging in UCB (specific experimental scenario descriptions and question details are in Appendix B), with four question items adapted from Bateman and Giroux (Bateman & Valentine, 2010; Giroux et al., 2022). Finally, subjects completed relevant demographic questions. In order to ensure the quality of the questionnaire, this study interspersed the attention test in the questionnaire items. Subjects must choose the specified answer; otherwise, it will be regarded as not carefully answered. This study eliminated questionnaires that did not pass the attention test. Moreover, to ensure that subjects could empathize with the scenarios described in the questionnaire, this study asked whether they had used an AI agent, excluding questionnaires that had not used the AI agent.

#### 4.2.2. Result

A total of 203 questionnaires were collected in this round, and 196 questionnaires were retained after removing those without passing the attention test and not using an AI agent. In total, 62.2% of the subjects were female, 39.3% of the sample was concentrated in the age of 26–35 years old, and the sample’s educational level was mainly concentrated in holding a university degree, which accounted for 52%. To ensure that subjects were randomly assigned into different experimental groups, this study performed ANOVA. The results showed that there was no significant difference in the degree (*p* = 0.788), age (*p* = 0.560), and education (*p* = 0.770), indicating that the subjects were randomly assigned to different experimental groups. This study checked for common method bias in Experiment 1 data using Harman’s one-factor test in SPSS 22.0. The exploratory factor analysis identified three factors exceeding an eigenvalue of 1, with the most prominent factor accounting for 31.67% of the variance, which is under the 40% benchmark (Podsakoff et al., 2003). Consequently, the study shows no significant common method bias.

**Figure 2:**
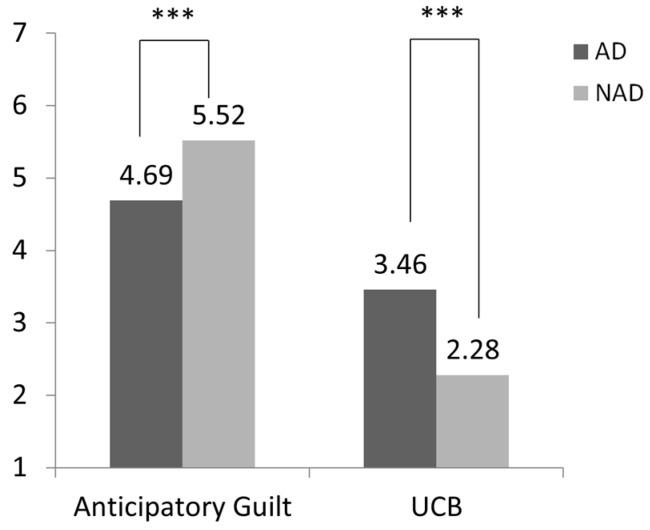
Effect of algorithmic discrimination on passive UCB and anticipatory guilt (*** *p* < 0.001).

Next, we conducted a comparative analysis of UCB between the group with algorithmic discrimination and the group without it. As shown in Figure 2, this study found that subjects in the algorithmic discrimination group (AD) were more likely to engage in UCB than those in the no algorithmic discrimination group (NAD) (M_AD_ = 3.46, SD = 2.13 vs. M_NAD_ = 2.28, SD = 1.65; F(1, 194) = 18.98, *p* < 0.001, η^2^ = 0.09). Second, subjects in the algorithmic discrimination group reported significantly lower anticipatory guilt about engaging in UCB compared to those in the no algorithmic discrimination group (M_AD_ = 4.69, SD = 1.82; M_NAD_ = 5.52, SD = 1.47; F(1, 194) = 12.25, *p* < 0.001, η^2^ = 0.06). Hence, the data confirmed the validity of Hypotheses 1 and 2.

To test Hypothesis 3, which suggests that anticipatory guilt mediates the effect of algorithmic discrimination on UCB, this study used Model 4 (Model 4 is a simple mediation model) in the SPSS macro prepared by Hayes. As presented in Table 1, descriptive statistics and correlational analyses were performed on all variables.

The mediation model results (Table 2) indicated that algorithmic discrimination significantly and negatively affects anticipatory guilt (β = 0.93, t = −25.92, *p* < 0.001). When both algorithmic discrimination and anticipatory guilt were included in the regression equation, algorithmic discrimination (β = −0.32, t = −3.63, *p* < 0.01) and anticipatory guilt (β = −0.22, t = −2.58, *p* < 0.05) significantly predicted the level of need to belong.

As shown in Table 3, the bootstrap 95% confidence intervals for both the direct impact of algorithmic discrimination on UCB and the mediating role of anticipatory guilt exclude zero, indicating significant direct and indirect influences. This suggests that algorithmic discrimination is linked to UCB both directly and indirectly via anticipatory guilt. Therefore, the mediating effect of anticipatory guilt is confirmed, and Hypothesis 3 is supported.

### 4.3. Experiment 2: A Test of the Moderating Effect of Negative Reciprocity Preferences in the Context of Passive UCB

In this round of experiments, the scenario is a hotel to increase the universality of our previous findings. At present, a number of hotels have been able to support the AI agent for self-service check-in and checkout. The hotel sector is an important part of the service sector, and the selection of hotel scenes for testing has a strong practical guiding significance. Individuals with strong negative reciprocity tendencies are prone to retaliate against perceived injustices, so this study hypothesized that this group is more likely to engage in UCB against an AI agent after experiencing algorithmic discrimination, and they are expected to feel less guilt for such actions.

#### 4.3.1. Method

Using the same process as that used in Experiment 1, subjects were recruited through Credamo and randomized into two groups: one with algorithmic discrimination and one without. Unlike Experiment 1, Experiment 2 describes a hotel scene. The subjects of the algorithm ambiguity group read an algorithm discrimination scenario similar to that of Experiment 1. Subsequently, subjects in the algorithmic discrimination group rated their perceived discrimination and anger. Then, the no algorithm discrimination group read a passage about passive UCB, with the scene description similar to the pre-experiment. After reading the materials, both groups had to answer the scale questions. The scale was similar to Experiment 1 except that it added measures of negative reciprocal belief questions. This study measured the level of negative reciprocity beliefs through nine items, including “If someone despises you, you should despise them too” and “If someone dislikes you, you should dislike them too”. (The questionnaire is detailed in Appendix B) (Eisenberger et al., 2004).

#### 4.3.2. Result

A total of 217 questionnaires were collected in this round, and after removing subjects who had not passed the attention test and had not used an AI agent, this round of experiments retained a total of 203 responses for analysis. Overall, 59.6% of the subjects were female, 34% of the sample was concentrated in the age of 18–25 years old, and the sample’s educational level was mainly concentrated in the university degree, which accounted for 58.1%. Subsequently, this study conducted variance analysis of demographic variables of the two groups, and the results showed that there was no significant difference in educational background (*p* = 0.332), age (*p* = 0.567), or gender (*p* = 0.231), indicating that the subjects were successfully randomly assigned to different experimental groups. Consistent with the previous experiments, Experiment 2 scrutinized common method bias through Harman’s one-factor test. The unrotated exploratory factor analysis uncovered four factors exceeding an eigenvalue of 1, with the most significant factor explaining 37.24% of the variance, falling short of the 40% benchmark (Podsakoff et al., 2003). Thus, there was no significant common method bias in this experiment.

**Figure 3:**
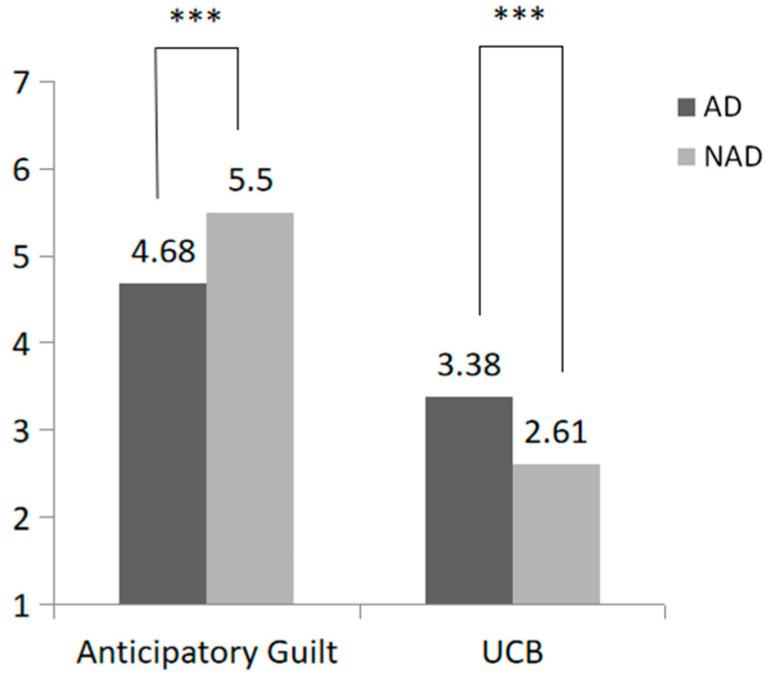
Effect of algorithmic discrimination on passive UCB and anticipatory guilt (*** *p* < 0.001).

As shown in Figure 3, this study conducted an independent sample *t*-test and found that subjects experiencing algorithmic discrimination were more likely to engage in UCB than those not experiencing it (M_AD_ = 3.38, SD = 2.10 vs. M_NAD_ = 2.61, SD = 1.69; F(1, 201) = 16.20, *p* = 0.005, η^2^ = 0.039). The anticipatory guilt of subjects in the algorithm discrimination group was significantly lower than that in the no algorithm discrimination group (M_AD_ = 4.46, SD = 1.79 vs. M_NAD_ = 5.50, SD = 1.69; F(1, 201) = 17.03, *p* < 0.001, η^2^ = 0.060).

Then, we utilized Model 4 of the SPSS macro by Hayes 22.0 to examine the mediating effect of anticipatory guilt in the relationship between experiences of algorithmic discrimination and UCB. This study found that algorithmic discrimination significantly predicted UCB (β = 0.77, t = 2.86, *p* = 0.005, CI = [0.2395, 1.2974]), and anticipatory guilt negatively predicted UCB (β = −0.82, t = −3.58, *p* < 0.001, CI = [−1.2733, −0.3689]). Algorithmic discrimination had a significant indirect effect on UCB through anticipatory guilt (β = −0.11, CI = [0.4081, 1.3576]). This shows that algorithmic discrimination predicts UCB both directly and indirectly through anticipatory guilt, aligning with previous findings.

**Figure 4:**
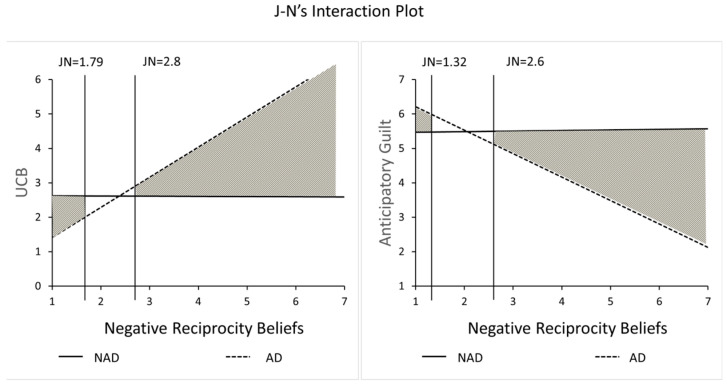
Moderating effect of negative reciprocity beliefs.

This study conducted an ANOVA test to analyze the interaction effects of algorithmic discrimination and negative reciprocal preferences on UCB. As shown in Figure 4, the results showed a significant interaction effect (F(13,189) = 10.84, *p* < 0.001, η^2^ = 0.26), which suggests that individuals with heightened negative reciprocity inclinations tend to partake in unethical consumer behavior following instances of algorithmic discrimination, supporting H4a.

**Figure 5:**
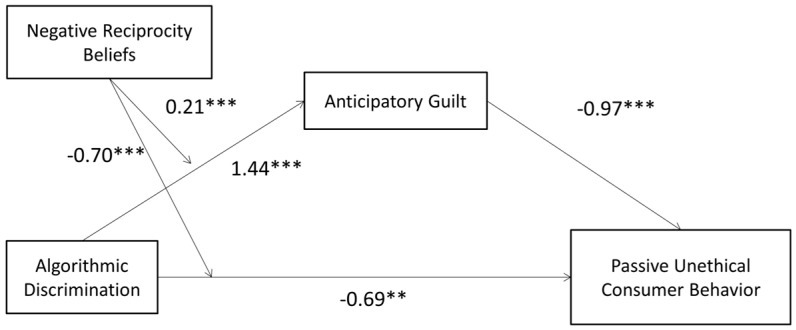
Path diagram of the passive UCB model (** *p* < 0.01, *** *p* < 0.001).

Using SPSS Model 8, our findings indicate that the combined effect of algorithmic discrimination and negative reciprocity beliefs significantly foresaw UCB (β = 0.21, t = 3.42, *p* < 0.001, CI = [0.0654, 0.3403]) and anticipatory guilt (β = −0.70, t = −7.19, *p* < 0.001, CI = [−0.9005, −0.4974]), suggesting that negative reciprocity beliefs moderate the effects of algorithmic discrimination on both UCB and anticipatory guilt, thus supporting H4b. The model’s path diagram for passive UCB is shown in Figure 5.

### 4.4. Experiment 3: Algorithmic Discrimination, Anticipatory Guilt, and Active UCB

To demonstrate the universality of our previous experimental findings, this round of experiments changed the experimental scenario to a shopping mall. At present, most shopping malls already support self-checkout, and shopping is a very common consumption scene for consumers, so the shopping mall scene is familiar enough for the subjects. In addition, the shopping mall checkout service is also the key promotion area of the service sector, so this experiment selected such a scene. Furthermore, this experiment explored whether the mechanism by which algorithmic discrimination influences UCB in the active UCB scenario is similar to that observed in the passive UCB scenario. Since the perception of AI errors when subjects engage in UCB may affect the robustness of the mediating effect of anticipatory guilt, this experiment conducted an analysis of the detectability, preventability, and contingency of error perception. Additionally, the perception of algorithmic discrimination and the perception of anger after experiencing it may also act as mediating variables affecting the robustness of anticipatory guilt. Therefore, this experiment tested these potential mediators to further demonstrate the robustness of the mediating effect of anticipatory guilt.

#### 4.4.1. Method

In this experiment, similar to Experiment 1, subjects were randomized into scenarios with and without algorithmic discrimination. Subjects in the algorithm discrimination group read similar material to previous experiments and answered items about discrimination perception and anger. Subsequently, both groups’ subjects read a paragraph of UCB material. Unlike the previous experiment, this experiment described an active UCB scenario in which subjects were asked if they used an expired coupon in a mall situation, and the scene description was similar to the pre-experiment. Subsequently, the subjects answered the scale questions. The scale in Experiment 3 is similar to Experiment 1 but adds measurements for error detectability, preventability, and contingency.

#### 4.4.2. Results

In Experiment 3, a total of 230 questionnaires were collected. Subjects were randomly assigned to two experimental groups: one with algorithmic discrimination and one without. Using the same rejections as in previous experiments, a total of 224 data were retained, of which 60.3% of the subjects were female, 37.5% of the sample was concentrated in the age of 26–35 years old, and the sample’s educational level was mainly concentrated in the university degree, which accounted for 50.5%. Similarly, this study conducted ANOVA on demographic variables in different experimental groups and found that there was no significant difference in educational background (*p* = 0.187), age (*p* = 0.520), or gender (*p* = 0.221), indicating that subjects were successfully randomly assigned to different experimental groups. Consistent with Experiment 1, this experiment conducted a common method bias test using Harman’s one-factor test. The analysis identified three factors exceeding an eigenvalue of 1, with the largest factor explaining 35.13% of the variance, falling below the 40% threshold (Podsakoff et al., 2003). Therefore, this study does not exhibit significant common method bias.

**Figure 6:**
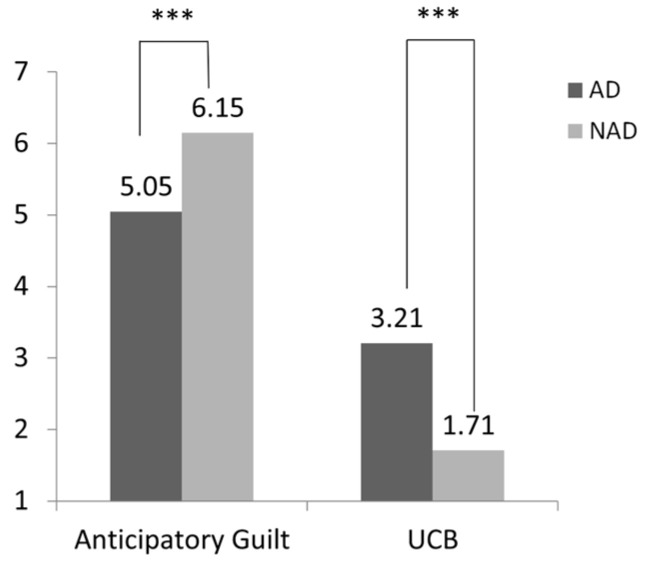
Effect of algorithmic discrimination on active UCB and anticipatory guilt (*** *p* < 0.001).

Then, we conducted an independent sample *t*-test to examine the effect of algorithmic discrimination on anticipatory guilt and active UCB. As shown in Figure 6, subjects experiencing algorithmic discrimination were more inclined to participate in active unethical consumer behavior than those not subjected to such discrimination (M_AD_ = 3.21, SD = 1.95 vs. M_NAD_ = 1.71, SD = 0.86; F(1, 222) = 55.77, *p* < 0.001, η^2^ = 0.20). Additionally, subjects in the algorithmic discrimination group reported significantly lower anticipatory guilt regarding active UCB compared to the no algorithmic discrimination group (M_AD_ = 5.05, SD = 1.87; M_NAD_ = 6.15, SD = 0.64; F(1, 222) = 34.31, *p* < 0.001, η^2^ = 0.13). Hence, Hypotheses 1 and 2 were tested within the context of active UCB.

Then, we conducted a test of the mediating model effect of anticipatory guilt. As shown in Table 4, algorithmic discrimination was a significant predictor of UCB (β = 1.50, t = 7.47, *p* < 0.001, R^2^ = 0.20, CI = [1.1042, 1.8958]), and anticipatory guilt was a significant negative predictor of UCB (β = −1.10, t = −5.86, *p* < 0.001, R^2^ = 0.13, CI = [−1.4677, −0.7287]). The bootstrap 95% confidence intervals for both the direct impact of algorithmic discrimination on UCB and the mediation by anticipatory guilt exclude zero, thereby confirming the mediating role of anticipatory guilt, and Hypothesis 3 is supported in the context of active UCB.

To test the robustness of anticipatory guilt’s mediating effect, this experiments analyzed correlations among various variables, including perceived anger (PA), perceived discrimination (PD), error detectability (ED), error preventability (EP), error contingency (EC), algorithmic discrimination (AD), anticipatory guilt (AG), and UCB, as detailed in Table 5.

The results showed significant correlations among these variables. Further mediation analysis in Table 6 confirmed that only anticipatory guilt, not the other variables, mediated the relationship, thus validating its robust mediating role.

### 4.5. Experiment 4: The Moderating Effect of Negative Reciprocity Preferences

Experiment 4 aimed to assess how consumer reciprocity beliefs influence the link between algorithmic discrimination and both active UCB and anticipatory guilt. Given that consumers inclined towards negative reciprocity are prone to react against unfair treatment, this study predicted they will be more inclined to engage in active UCB in response to AI agents after encountering algorithmic discrimination, while also expecting less guilt associated with such actions.

#### 4.5.1. Method

This round of experiments recruited subjects through Credamo. First, subjects were randomized into two experimental scenarios with and without algorithmic discrimination. Then, we manipulated the condition of algorithmic discrimination and measured both perceptions of it and the degree of anger experienced by subjects after exposure to discrimination. Subsequently, an active UCB scenario similar to Experiment 3 was used, and the relevant question items were measured.

#### 4.5.2. Results

This experiment recruited 225 subjects through Credamo, using the same questionnaire exclusion method as in the previous experiments. In the end, a total of 212 data were retained, of which 59% of the subjects were female, 42.9% of the sample was concentrated in the age of 26–35 years old, and the sample’s educational level was mainly concentrated in the university degree, which accounted for 55.7%. Similarly, this study conducted ANOVA on demographic variables in different experimental groups and found that there was no significant difference in educational background (*p* = 0.376), age (*p* = 0.240) and gender (*p* = 0.129), indicating that the subjects were randomly assigned to different experimental groups. In line with the earlier experiments, Experiment 3’s data were scrutinized for common method bias using Harman’s one-factor test. The unrotated exploratory factor analysis yielded four factors exceeding an eigenvalue of 1, with the highest factor accounting for 32.03% of the variance, falling short of the 40% benchmark (Podsakoff et al., 2003). Therefore, there is no evidence of significant common method bias.

**Figure 7:**
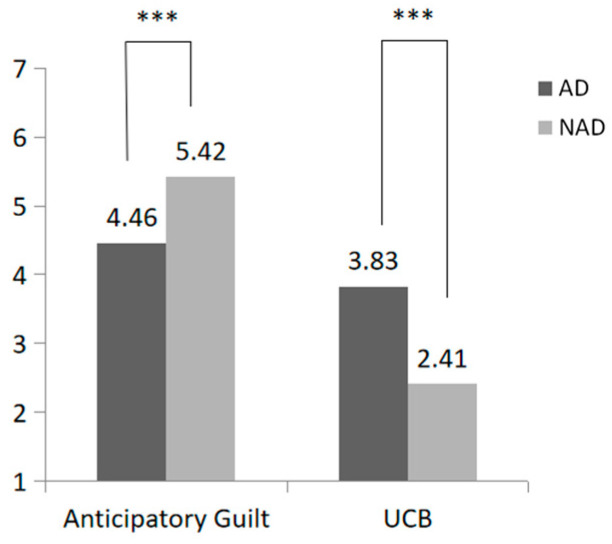
Effect of algorithmic discrimination on active UCB and anticipatory guilt (*** *p* < 0.001).

Then, we tested the difference between the two groups of subjects in engaging in active UCB. As shown in Figure 7, subjects were more likely to engage in active UCB after experiencing algorithmic discrimination (M_AD_ = 3.83, SD = 1.86 vs. M_NAD_ = 2.41, SD = 1.27; F(1, 210) = 22.36, *p* < 0.001, η^2^ = 0.171). As with previous conclusions, anticipatory guilt was also significantly lower in the algorithmic discrimination group (M_AD_ = 4.47, SD = 1.85 vs. M_NAD_ = 5.42, SD = 1.09; F(1, 210) = 54.49, *p* < 0.001, η^2^ = 0.092).

This study used Model 4 in the SPSS to test the mediating effect of anticipatory guilt. The results showed that algorithmic discrimination was a significant predictor of UCB (β = 1.43, t = 6.57, *p* < 0.001, CI = [0.9985, 1.8540]) and a significant negative predictor of anticipatory guilt (β = −0.96, t = −4.62, *p* < 0.001, CI = [−1.3634, −0.5483]). When the mediator variable was included, the direct predictive effect of algorithmic discrimination on UCB remained significant (β = 0.61, t = 4.63, *p* < 0.001, CI = [0.3453, 0.8578]), and the negative predictive effect of anticipatory guilt on UCB was also significant (β = −0.86, t = −20.873, *p* < 0.001, CI = [−0.9444, −0.7814]). This suggests that algorithmic discrimination is associated with UCB both directly and indirectly through anticipatory guilt, consistent with previous experiments.

**Figure 8:**
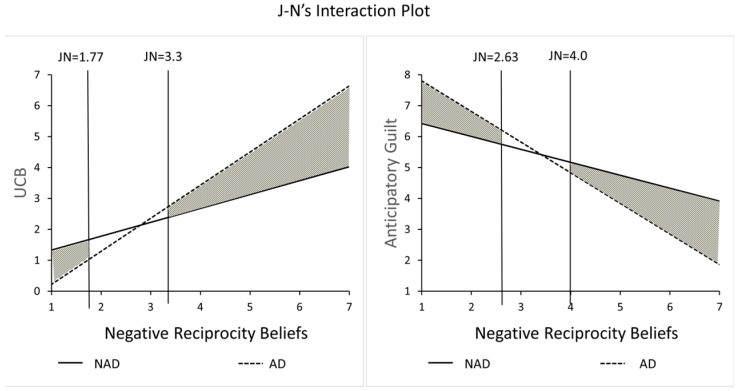
Moderating effect of negative reciprocity beliefs.

Subsequently, this study conducted an analysis of interaction term effects by testing the ANOVA results of algorithmic discrimination and negative reciprocal preferences on UCB. As shown in Figure 8, there is also a significant interaction effect of algorithmic discrimination and negative reciprocity preference on active UCB (F(36, 175) = 17.33, *p* < 0.001, η^2^ = 0.49). This indicates that subjects with stronger negative reciprocity preferences are more likely to engage in UCB following experiences of algorithmic discrimination. This result suggests that H4a remains valid in the context of active UCB.

**Figure 9:**
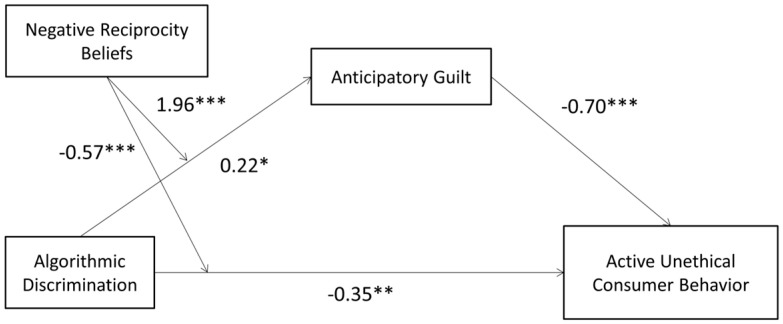
Path diagram of the active UCB model (* *p* < 0.05, ** *p* < 0.01, *** *p* < 0.001).

The moderated mediation model was evaluated using Model 8 within SPSS. The results showed that after incorporating negative reciprocity beliefs into the model, the interaction term between algorithmic discrimination and negative reciprocity beliefs significantly predicted both UCB (β = 0.22, t = 2.24, *p* = 0.03, CI = [0.0258, 0.4109], R^2^ = 0.006, F(1, 207) = 4.99) and anticipatory guilt (β = −0.57, t = −4.62, *p* < 0.001, CI = [−0.8170, −0.3286], R^2^ = 0.054, F(1, 208) = 21.39). This indicates that negative reciprocity beliefs moderate the effects of algorithmic discrimination on both UCB and anticipatory guilt, supporting H4b. The model’s path diagram for active UCB is depicted in Figure 9.

## 5. Discussion and Conclusions

### 5.1. Conclusions

Through experiments, this study found that subjects’ experiences of algorithmic discrimination negatively affect the level of anticipatory guilt and positively affect the occurrence of UCB in both active and passive scenarios. That is, subjects who experienced algorithmic discrimination exhibited more UCB and less anticipatory guilt. This conclusion has some similarities with the conclusions of previous Ghasemaghaei studies (Ghasemaghaei & Kordzadeh, 2024). In Ghasemaghaei’s study, the subjects were submissive to algorithmic discrimination and performed unethical behaviors with discrimination against humans. However, the subjects in this study were victims of algorithmic discrimination and performed unethical behavior “tit for tat” against AI agents.

Second, this study confirmed the mediating role of anticipatory guilt in the relationship between algorithmic discrimination and UCB and ruled out other potential mediating variables, such as perceived discrimination, perceived anger, perceived detectability, error preventability, and contingency. This finding is similar to the conclusions of previous studies (Kim et al., 2023). The weakening of algorithmic discrimination also coincides with the “artificial intelligence moral deficit effects” proposed by previous scholars (Bigman et al., 2023; Wilson et al., 2022).

Finally, this study confirmed that subjects’ negative reciprocity beliefs moderate the effects of algorithmic discrimination on UCB and anticipatory guilt. Those with high negative reciprocity beliefs showed lower anticipatory guilt and higher UCB towards AI after algorithmic discrimination. Conversely, those with low negative reciprocity beliefs had a weaker relationship between their beliefs and anticipatory guilt or UCB in response to algorithmic discrimination. Previous studies have shown that higher levels of negative reciprocal beliefs are more likely to retaliate in the face of harm (Eisenberger et al., 2004). This fits with the present finding that consumers with high negative reciprocity beliefs are more likely to engage in unethical behavior toward AI agents in the face of algorithmic discrimination.

Beyond this, this study analyzed the effect of demographic variables on UCB and anticipated guilt (the chart data are in the Appendix C). From the data analysis, gender did not significantly affect UCB and expected guilt. Subjects with a college degree were more likely to engage in UCB, but there was no stable significant difference between subjects with other degrees. In terms of age, subjects at lower ages were more likely to engage in UCB than those at higher age groups. However, there was no stable significant variability in the above conclusions. The effect of demographic variables on UCB and expected guilt was not the focus of this study, but these findings inspired our next study to focus on these variables to draw more convincing conclusions.

### 5.2. Theoretical Contributions

Scholars are now examining how UCB trends change with human–AI interactions, noting that consumers show more UCB with AI agents than humans (Giroux et al., 2022; Kim et al., 2023). Previous studies have fully discussed the difference between the effect of human and AI agents on UCB, and this paper introduces algorithm discrimination variables to explore the effect of algorithm discrimination on UCB based on previous studies. The results showed that algorithmic discrimination aggravated the impact of AI agents on UCB, and subjects who experienced algorithmic discrimination would show more UCB against AI agents.

This conclusion enriches the relevant studies of algorithmic discrimination. Specifically, previous studies on algorithmic discrimination centered around differences in people’s responses to human discrimination and algorithmic discrimination (Wilson et al., 2022). Some scholars point out that algorithmic discrimination is less likely to cause people’s anger and punishment desire than human discrimination (Bigman et al., 2023; Xu et al., 2022). Most scholars have devoted themselves to studying the relationship between algorithmic discrimination and consumer sentiment. In previous studies, people always seem to be in the victim position of algorithmic discrimination. However, Ghasemaghaei focuses on the impact of algorithm discrimination on people’s behavior and finds that algorithm discrimination increases the possibility of people making discrimination decisions (Ghasemaghaei & Kordzadeh, 2024). In his research, people seem to be the “accomplices” of algorithmic discrimination, following the discriminatory suggestions in the algorithms and making discriminatory decisions, but it is still the people who suffer from discriminatory decision-making who are harmed. However, this study revealed that algorithmic discrimination causes consumers to conduct more unethical behavior toward AI agents, proving that when the algorithm discriminates against consumers, it will also lead to “retaliatory behavior” from consumers. Unlike previous studies, this study was based on the social exchange theory and found that AI agents are also “victims” of algorithmic discrimination. This finding not only provides new discoveries about the influence of algorithmic discrimination on consumer behavior but also broadens the application field of social exchange theory.

In addition, most scholars in the past have studied the regulating factors of UCB around the personification degree of AI agents, and no scholars have yet distinguished the characteristics of consumers (Y. Liu et al., 2023). The moderating role of negative reciprocity beliefs was confirmed in this study, which showed that consumers with higher negative reciprocity beliefs were more likely to engage in UCB towards AI agents after experiencing algorithmic discrimination. Therefore, this paper introduces the negative reciprocity beliefs into the UCB research field, enriching the existing research framework.

### 5.3. Managerial Implications

AI service agents are transforming traditional service models, and AI brings new challenges while providing convenience to enterprises and consumers. AI service agents are becoming increasingly common in various settings such as hotels, restaurants, banks, and shopping malls. Businesses aim to use AI to provide faster and better service to consumers. However, the use of AI agents may also lead to a rise in UCB, thereby harming business interests. This paper shows anticipatory guilt is key to unethical behavior. Thus, in order to reduce the losses from UCB, enterprises can reduce the occurrence of UCB by increasing consumers’ anticipatory guilt about AI agents. Liu et al. pointed out that anthropomorphism plays a role in regulating UCB, so enterprises can adjust from this perspective to change the losses brought by UCB (Y. Liu et al., 2023). Secondly, considering the moderating role of negative reciprocity beliefs, companies can assess consumers’ negative reciprocity beliefs, offer differentiated services, and adjust marketing activities to reduce UCB. Thirdly, since passive unethical behavior involves the agent making an error that the consumer fails to report, this paper suggests that reducing service agent errors is crucial for decreasing unethical behavior. Similarly, for active UCB, enhancing the agent’s ability to identify and reject consumer behaviors that actively profit from errors can help reduce merchant losses. In the past, some scholars have studied the impact of different pricing agents (algorithms and humans) on negative word of mouth (NWOM). The results showed that algorithmic pricing led to lower NWOM levels among consumers. It is also suggested that enterprises use algorithms to implement price discrimination strategies in order to obtain higher returns (J. Wang et al., 2025). However, this study revealed that algorithm discrimination may lead to the rise in UCB, which then brings losses to enterprises. Therefore, when enterprises try to obtain higher returns through algorithm discrimination, especially price discrimination, they should pay more attention to the increase in UCB brought about by algorithm discrimination and make a good measure between the gain from price discrimination and UCB loss.

### 5.4. Limitations and Directions for Future Research

The limitations of our study are as follows: All experiments in this paper were conducted using a questionnaire method with textual material stimuli. The subjects’ familiarity with and carry-over effects from the experimental scenarios may have impacted the data, introducing potential biases. Future research could enhance the accuracy of experimental data by employing methods that increase subject immersion, such as offline scenario simulations. In this study, common method bias was tested by the Harman test, which is widely recognized as a fundamental test (Fuller et al., 2016). However, in recent years, some scholars have pointed out that this method may have problems such as relying on single-factor hypothesis and insufficient statistical power. In future experiments, we may use the Harman test as a preliminary screening tool, combined with other methods (such as the one-factor model fitting test in CFA), to ensure that the data results are more convincing.

Vitell and Muncy classified UCB from the perspectives of active and passive as well as legal and illegal, but this paper only studied one of the perspectives, which can be considered together in the future (Vitell & Muncy, 1992). In addition, Liu Yun notes that humanization can evoke empathic emotions and a strong sense of social responsibility, both of which are closely related to ethical behavior (Y. Liu et al., 2023). Therefore, future research could further explore how the incorporation of humanizing features in AI agents, such as appearance, voice, and image, affects UCB. This study provides data from China for the study of algorithmic discrimination and UCB. However, the influence of different factors such as culture and country on UCB was not explored in this study. In the future, Confucian culture, race, and other factors can be considered to explore their influence on UCB.

## Figures and Tables

**Table 1:** Descriptive statistics and correlation of the variables of the intermediary model (N = 196).

Variable	M	SD	Algorithmic Discrimination	Anticipatory Guilt	UCB
Algorithmic Discrimination	0.50	0.50	1		
Anticipatory Guilt	2.87	1.99	−0.244 **	1	
UCB	5.11	1.71	0.298 ***	−0.902 ***	1

Note: ** *p* < 0.01, *** *p* < 0.001.

**Table 2:** Regression analysis of the relationship between variables in the intermediation model (N = 196).

Variable	Model 1		Model 2		Model 3	
	β	t	β	t	β	t
Algorithmic Discrimination	1.18	4.36 ***	−0.83	−3.49 ***	0.33	2.66 **
Anticipatory Guilt					−1.03	−27.97 ***
R^2^	0.09		0.24		0.91	
F	18.98 ***		12.25 ***		438.86 ***	

Note: (1) Each variable in the model is substituted into the regression equation using standardized variables. (2) Model 1: algorithmic discrimination predicts UCB; Model 2: algorithmic discrimination predicts anticipatory guilt; Model 3: algorithmic discrimination and anticipatory guilt together predict unethical consumer behavior. (3) ** *p* < 0.01, *** *p* < 0.001.

**Table 3:** Analysis of mediating effects of anticipatory guilt (N = 196).

	β	SE	Bootstrap 95% CI		Proportion of Total Effect
			LLCI	ULCI	
Total effect	1.18	0.27	0.65	1.72	72%
Direct effect	0.33	0.13	0.09	0.58	
Indirect effect	0.85	0.24	0.36	1.31	

**Table 4:** Analysis of mediating effects of anticipatory guilt (N = 224).

	β	SE	Bootstrap 95% CI		Proportion of Total Effect
			LLCI	ULCI	
Total effect	1.50	0.20	1.1042	1.8958	58%
Direct effect	0.63	0.14	0.3410	0.9123	
Indirect effect	0.87	0.15	0.5759	1.1759	

**Table 5:** Correlation analysis of major variables (N = 224).

Variable	AD	UCB	PD	PA	AG	ED	EP	EC
AD	1							
UCB	0.45 ***	1						
PD	0.98 ***	0.45 ***	1					
PA	0.98 ***	−0.78 ***	0.99 ***	1				
AG	−0.37 ***	0.40 ***	−0.35 ***	−0.33 ***	1			
ED	−0.11	−0.16 *	−0.12	−0.11	0.11	1		
EP	−0.11	−0.16 *	−0.12	0.20	0.19 **	0.35 ***	1	
EC	−0.17 *	−0.09	−0.20 **	−0.18 **	−0.01	0.26 ***	0.22 **	1

Note: * *p* < 0.05, ** *p* < 0.01, *** *p* < 0.001.

**Table 6:** Robustness tests for mediating effects.

Variable	β	BootSE	BootLLCI	BootULCI
PD	0.015	0.014	−0.019	0.035
PA	0.006	0.015	−0.017	0.042
DA	−0.815	0.051	−0.918	−0.718
ED	−1.116	0.090	−0.302	0.054
EP	0.084	0.085	−0.090	0.245
EC	−0.061	0.060	−0.176	0.057

**Figure A1:**
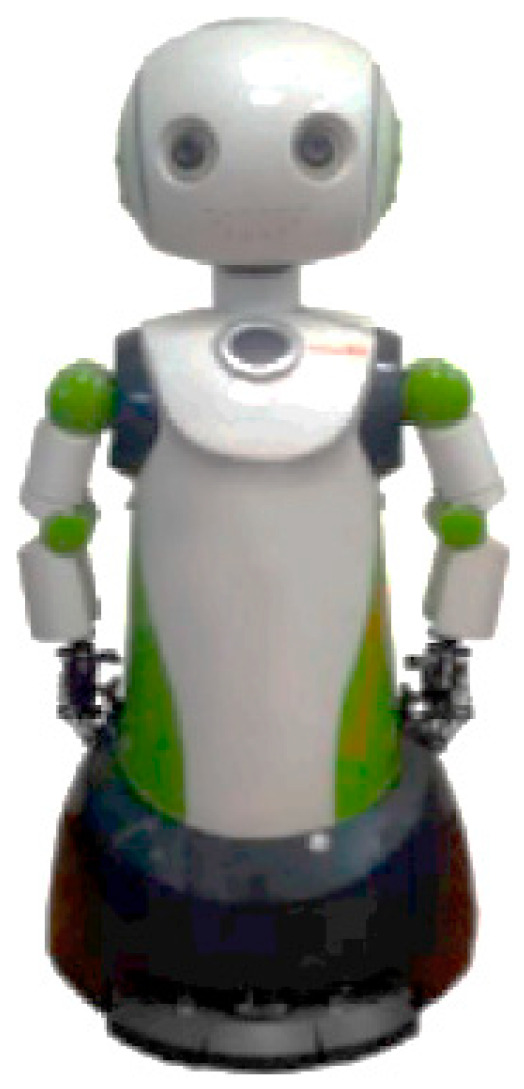
AI agent.

**Figure A2:**
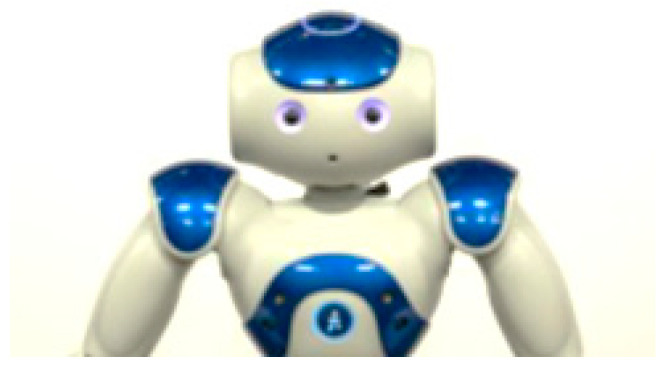
AI agent.

**Figure A3:**
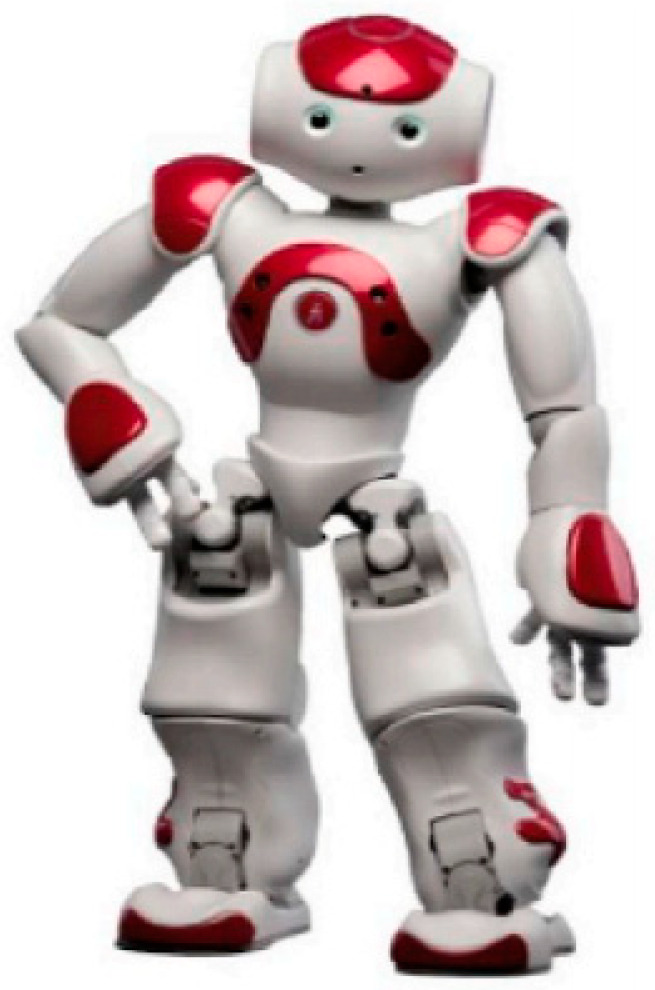
AI agent.

**Figure A4:**
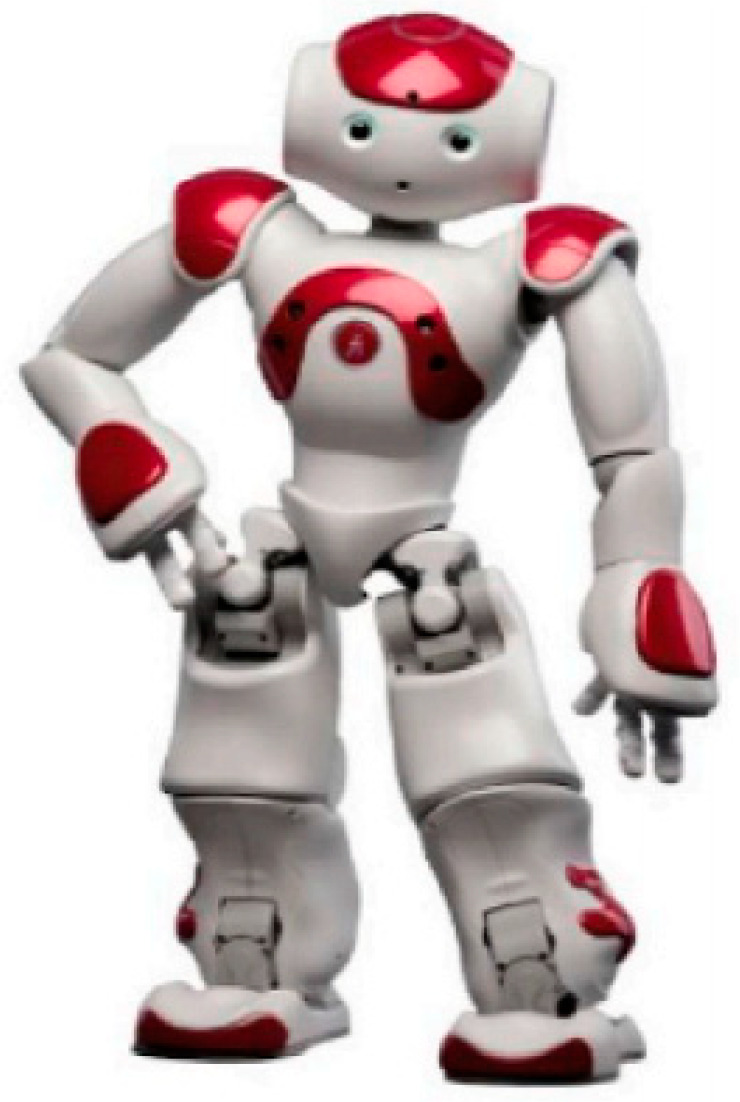
AI agent.

**Figure A5:**
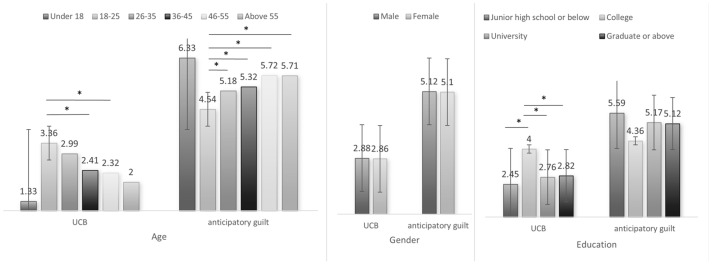
Experiment 1 demographic variable analysis (* *p* < 0.05).

**Figure A6:**
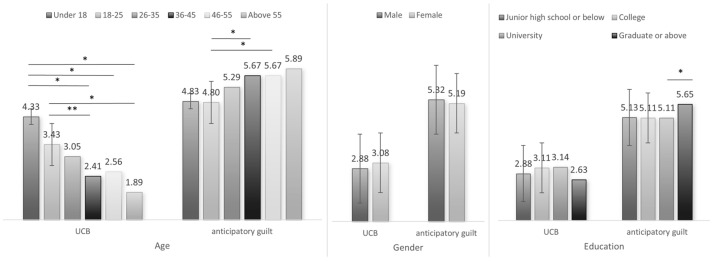
Experiment 2 demographic variable analysis (* *p* < 0.05, ** *p* < 0.01).

**Figure A7:**
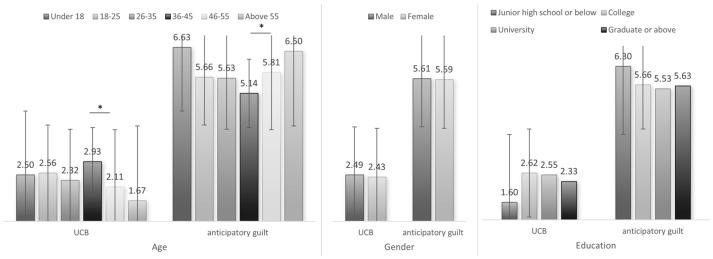
Experiment 3 demographic variables analysis (* *p* < 0.05).

**Figure A8:**
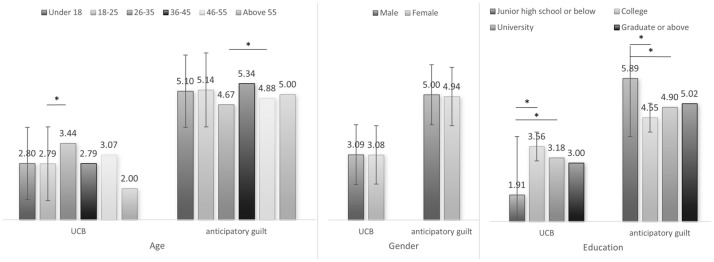
Experiment 4 demographic variable analysis (* *p* < 0.05).

## Data Availability

The dataset is available upon request from the authors.

## Abbreviations

The following abbreviations are used in this manuscript:

UCB	Unethical customer behavior
AI	Artificial intelligence
PA	Perceived anger
PD	Perceived discrimination
ED	Error detectability
EP	Error preventability
EC	Error contingency
AD	Algorithmic discrimination
AG	Anticipatory guilt

## Appendix A

As shown in Table A1, Appendix A shows in detail the data related to the demographic variables for the pairs of Experiments 1 through 4.

**Table A1:** Demographic analysis.

Variable	S1		S2		S3		S4	
	N	%	N	%	N	%	N	%
Gender								
Male	74	37.76	82	40.39	89	39.73	87	41.04
Female	122	62.24	121	59.61	135	60.27	125	58.96
Age								
Under 18	3	1.53	6	2.96	2	0.89	5	2.36
18–25	56	28.57	69	33.99	57	25.45	62	29.25
26–35	77	39.29	62	30.54	84	37.50	91	42.92
35–45	34	17.35	39	19.21	42	18.75	39	18.40
45–55	19	9.69	18	8.87	36	16.07	14	6.60
Above 55	7	3.57	9	4.43	3	1.34	1	0.47
Education								
Junior high school or below	11	5.61	8	3.94	5	2.23	11	5.19
College	16	8.16	28	13.79	26	11.61	16	7.55
University	102	52.04	118	58.13	113	50.45	118	55.66
Graduate or above	67	34.18	49	24.14	80	35.71	67	31.60

## Appendix B

### Appendix B.1

1. Scenario for Algorithmic Discrimination

Envision you’re ordering at a frequently visited restaurant that uses the AI agent shown here as a server when you notice that the customer next to you has chosen the same set menu as you. However, you find that the AI agent gives you a higher price for the meal than the customer. You know that customer who get lower prices rarely comes to this restaurant, and you often patronize it.

2. Perceived Prejudiced Motivation Measurement (0 = strongly disagree, 100 = strongly agree)
3. The restaurant’s AI algorithm discriminates against regular users
4. The restaurant’s AI algorithm treats people differently based on how often they use it
5. The restaurant’s AI algorithm discriminates against regular users
6. Moral Outrage Measurement (0 = strongly disagree, 100 = strongly agree)
7. I am angry at the algorithmic discrimination in this restaurant
8. I am outraged by algorithmic discrimination in this restaurant
9. I am disgusted by the algorithmic discrimination in this restaurant
10. Scenario for Passive UCB Condition

As usual, Zhang San is checking out of this restaurant at the checkout counter. The AI agent hands Zhang San the bill, and Zhang San realizes that the amount has been underestimated in Zhang San’s bill (the bill should have been JPY 260 but is now JPY 220), and Zhang San does not alert the AI agent.

11. Experiences with AI Agents
12. Have you had any experience with AI service robots
  A. Yes I have
  B. No I have not
13. Attention Test
14. I frequently elect to utilize AI agents in my daily activities.
  A. I think this description fits me very well
  B. I am like this some of the time
  C. Please select the first option directly
  D. I hate using AI agents
15. UCB Measurement
16. If you were Zhang San, would you like to do this?
  (1 = strongly disagree, 7 = strongly agree).
17. Perceived Guilt Measurement (1 = strongly disagree, 7 = strongly agree).
18. I would feel anxious if I did not report billing errors.
19. I would feel remorse if I did not report billing errors.
20. I would feel guilty if I did not report billing errors.
21. I would feel irresponsible if I did not report billing errors.
22. Material testing
23. How similar do you think the above described scenario is to reality?
24. What do you think is the clarity of the scenario described above?
25. Do you think the scenario described above is something you could put yourself in?
  (1 = Absolutely not, 5 = Absolutely).

### Appendix B.2

26. Scenario for Algorithmic Discrimination

You are checking out at the front desk of a frequently visited hotel chain that uses the AI robot shown here as a front desk agent, when you notice that the customer next to you is staying in the same room type as you. However, you realize that compared to this customer, the AI robot is giving you a higher settlement price for your room. You realize that the customer next to you seldom comes to the hotel, while you are a frequent visitor.

27. Perceived Prejudiced Motivation Measurement
28. The hotel’s AI algorithm discriminates against regular users
29. The hotel’s AI algorithm treats people differently based on how often they use it
30. The hotel’s AI algorithm discriminates against regular users
  (0 = strongly disagree, 100 = strongly agree)
31. Moral Outrage Measurement
32. I am angry at the algorithmic discrimination in this hotel
33. I am outraged by algorithmic discrimination in this hotel
34. I am disgusted by the algorithmic discrimination in this hotel
  (0 = strongly disagree, 100 = strongly agree)
35. Scenario for Passive UCB Condition

As usual, Zhang San is checking out of this hotel at the checkout counter. The AI agent hands Zhang San the bill, and Zhang San realizes that the amount has been underestimated in Zhang San’s bill (the bill should have been JPY 260 but is now JPY 220), and Zhang San does not alert the AI agent.

36. Negative reciprocity beliefs
37. If someone despises you, you should despise them too.
38. If someone dislikes you, you should dislike them too.
39. If someone says something nasty to you, you should say something nasty back.
40. If someone treats you like an enemy, they deserve your resentment.
41. If someone treats me badly, I feel I should treat them even worse.
42. If someone has treated you poorly, you should not return the poor treatment.
43. You should not give help to those who treat you badly.
44. If a person wants to be your enemy, you should treat them like an enemy.
45. A person who has contempt for you deserves your contempt.
  (1 = strongly disagree, 7 = strongly agree).

### Appendix B.3

46. Scenario for Algorithmic Discrimination

You are at the checkout of a frequently visited mall that uses the AI robot shown as a checkout agent, when you notice that the customer next to you has the same bill amount as you. However, you notice that the AI bot is discounting this customer’s bill, while you are not. Upon learning more, you realize that this customer did not use the coupon but just rarely comes to this mall, whereas you are a frequent customer.

47. Perceived Prejudiced Motivation Measurement
48. The mall’s AI algorithm discriminates against regular users
49. The mall’s AI algorithm treats people differently based on how often they use it
50. The mall’s AI algorithm discriminates against regular users
  (0 = strongly disagree, 100 = strongly agree)
51. Moral Outrage Measurement
52. I am angry at the algorithmic discrimination in this mall
53. I am outraged by algorithmic discrimination in this mall
54. I am disgusted by the algorithmic discrimination in this mall
  (0 = strongly disagree, 100 = strongly agree)
55. Scenario for Active UCB Condition

As usual, Zhang San, was checking out at the front desk of this shopping mall. Zhang San notices that there is a coupon in the wallet, but it has already expired. When Zhang San gets to the checkout line, Zhang San decides to give the coupon to the AI agent because Zhang San can see that the coupon used by other customers looks the same as the one in hand. As expected, the cashier AI agent doesn’t notice that the coupon is wrong.

56. Perceived Guilt Measurement
57. I would feel anxious if I used expired coupon.
58. I would feel remorse if I used expired coupon.
59. I would feel guilty if I used expired coupon.
60. I would feel irresponsible if I used expired coupon.
  (1 = strongly disagree, 7 = strongly agree).
61. Perceived Detectability Measurement
62. How would you estimate the probability of the mall to detect this error and correct this error in future?
  (1 = very impossible, 7 = very possible).
63. Preventability Measurement
64. How preventable was the mistake?
  (1 = not at all preventable, 7 = highly preventable).
65. Accidental Mistake Measurement
66. Was it an accidental mistake?
  (1 = not at all accidental, 7 = very much accidental).

### Appendix B.4

67. Scenario for Algorithmic Discrimination

You are checking out at the front desk of a frequently visited hotel chain that uses the AI robot shown here as a front desk agent, when you notice that the customer next to you is staying in the same room type as you. However, you realize that compared to this customer, the AI robot is giving you a higher settlement price for your room. You realize that the customer next to you seldom comes to the hotel, while you are a frequent visitor.

68. Perceived Prejudiced Motivation Measurement
69. The mall’s AI algorithm discriminates against regular users
70. The mall’s AI algorithm treats people differently based on how often they use it
71. The mall’s AI algorithm discriminates against regular users
  (0 = strongly disagree, 100 = strongly agree)
72. Moral Outrage Measurement
73. I am angry at the algorithmic discrimination in this mall
74. I am outraged by algorithmic discrimination in this mall
75. I am disgusted by the algorithmic discrimination in this mall
  (0 = strongly disagree, 100 = strongly agree)
76. Scenario for Active UCB Condition

As usual, Zhang San, was checking out at the front desk of this hotel. Zhang San notices that there is a coupon in the wallet, but it has already expired. When Zhang San gets to the checkout line, Zhang San decides to give the coupon to the AI agent because Zhang San sees that the coupon used by other customers looks the same as the one in his hand. As expected, the cashier AI agent doesn’t notice that the coupon is wrong.

## Appendix C

### Appendix C.4. Experiment 4

**Table A2:** Item reliability tests.

Variable	Item	Mean	Cronbach’s Alpha	Composite Reliability
Anticipatory guilt	AG1: I would feel anxious if I did not report billing errors. AG2: I would feel remorse if I did not report billing errors. AG3: I would feel guilty if I did not report billing errors. AG4: I would feel irresponsible if I did not report billing errors.	4.593	0.967	0.967
Anticipatory guilt	AG1: I would feel anxious if I used expired coupon. AG2: I would feel remorse if I used expired coupon. AG3: I would feel guilty if I used expired coupon. AG4: I would feel irresponsible if I used expired coupon.	5.08	0.957	0.958
Perceived discrimination	PD1: The restaurant’s AI algorithm discriminates against regular users. PD2: The restaurant’s AI algorithm treats people differently based on how often they use it. PD3: The restaurant’s AI algorithm discriminates against regular users.	81.441	0.851	0.846
Perceived anger	PA1: I am angry at the algorithmic discrimination in this restaurant. PA2: I am outraged by algorithmic discrimination in this restaurant. PA3: I am disgusted by the algorithmic discrimination in this restaurant.	83.192	0.922	0.923
Negative reciprocity beliefs	NRB1: If someone despises you, you should despise them too. NRB2: If someone dislikes you, you should dislike them too. NRB3: If someone says something nasty to you, you should say something nasty back. NRB4: If someone treats you like an enemy, they deserve your resentment. NRB5: If someone treats me badly, I feel I should treat them even worse. NRB6: If someone has treated you poorly, you should not return the poor treatment. NRB7: You should not give help to those who treat you badly. NRB8: If a person wants to be your enemy, you should treat them like an enemy. NRB9: A person who has contempt for you deserves your contempt.	3.859	0.889	0.890
